# A Live Issue in Medical Economics

**Published:** 1915-04

**Authors:** 


### A Live Issue in Medical Economics.

Some months ago, we alluded to the fact that, in spite of favorable tendencies to decrease the production of physicians by educational requirements, the overcrowding of the profession was a serious matter, especially in cities, while many small villages felt the need of a physician. A number of candid letters have emphasized a point whose importance we had not fully realized: While the ratio of population to physicians has not decreased and has, if anything increased, sanitary and hygienic knowledge has actually produced a notable diminution of disease, estimated at something like 25%. We have already mentioned the very considerable number of official positions open to physicians. Without having succeeded in gathering accurate statistics, probably there are at least 10,000 such positions available in the country that offer living salaries. This corresponds to about 8% of the profession, actually in practice for the lists of physicians are padded with those more or less definitely retired, not so much by age as by occupation in other industries. Probably for every 100 physicians listed, there are at least ten more not listed at all and of the hundred, at least ten are not in actual practice to a degree economically considerable—and by the last statement we do not allude to beginners whose practice has not been established but who are potentially active competitive factors. The important point that we have overlooked is that the approximately 8% removed from direct competition by holding official appointments with the nation, state, county, city, etc., are indirectly even more important competitive factors since, on the whole, they do an amount of work disproportionate to their salaries, as compared with average professional earnings and, in so far as they are engaged in sanitary work, they actually prevent a vastly larger amount of sickness than would correspond to the direct average competition. It ought not to be necessary to say that we would not have this otherwise but, at the same time, the fact must be clearly grasped in attempting to solve the economic problem.

As a matter of prophylaxis, it must be kept before the minds of medical sociologists that the ratio of white to red corpuscles which, a generation ago, represented the normal ratio of physicians to population, on a reasonably satisfactory though not easy economic basis, is no longer tenable. On the other hand, we have opposed (1) the establishment of an arbitrary and unreasonable requirement of the prospective physician and (2) the deliberate killing off of medical schools otherwise worthy but financially weak. This policy is due to the conception that, however pressing an economic problem may be, the welfare of the whole medical body does not justify marked injustice to the individual candidate or institution. A candid presentation of the existing facts and a gradual and reasonable elevation of requirements will eventually secure a readjustment of the balance of supply and demand.

Meantime, the urgency of the economic situation has been impressed upon us by several letters from physicians more or less acutely in need of means of support. None of these are subscribers, residents of western New York nor in search of locations for private practice. For these reasons, personal acquaintance and familiarity with local conditions do not bear on the situation.

We ask our advertisers who have employment for physicians, our subscribers who may be in need of assistants or who may have information as to localities needing a physician or as to available positions of any kind, to bear in mind these requests. We shall be pleased to act as a clearing house and to respect all confidences.

---

NOTICE: The BUFFALO MEDICAL JOURNAL will gladly co-operate with any affiliated profession, or institution, or society or local unit which desires the advantages of a publication without the expense and labor involved in the maintenance of a separate organ. The general plan will be about as follows: Additional space will be furnished at the prorata cost of printing. The department added will be entirely under the control of the organization concerned, subject only to such restrictions as are obvious. The JOURNAL will profit only indirectly, through the increase of subscriptions and publicity. If the department is wanted only for part of the issues, as for example, a college session, at quarterly intervals, etc., arrangements will be made for partial subscriptions, if so desired. Readers are requested to bear this notice in mind for future reference.

